# The Burden of Disease and Health Care Use among Pertussis Cases in School Aged Children and Adults in England and Wales; A Patient Survey

**DOI:** 10.1371/journal.pone.0111807

**Published:** 2014-11-25

**Authors:** Albert Jan van Hoek, Helen Campbell, Nick Andrews, Mariza Vasconcelos, Gayatri Amirthalingam, Elizabeth Miller

**Affiliations:** 1 Immunisation, Hepatitis and Blood Safety Department, Public Health England, London, England; 2 Statistics and modelling unit, Public Health England, London, England; University of Melbourne, Australia

## Abstract

**Background:**

In 2011–2012 a large pertussis outbreak occurred in England. This provided an opportunity to estimate the disease burden in those aged 5 years and over. As pertussis is likely to be under reported both laboratory-confirmed and non-confirmed cases were included.

**Methods:**

Laboratory-confirmed cases of pertussis, as well as their coughing but non-confirmed household members, were sent a questionnaire that collected information on clinical features and quality of life for the most severe day of disease and the day the patient filled in the questionnaire. The EuroQol-5 dimension questionnaire (EQ-5D) was used to evaluate quality of life. The duration of symptoms was obtained by contacting the patient every two weeks until symptoms stopped.

**Results:**

Data for 535 (out of 1262) laboratory confirmed pertussis patients and 44 (out of 140) coughing household contacts was available for analysis. On the most severe day, 56% of laboratory-confirmed cases reported they had 20+ more paroxysms, 58% reported they had a severe cough and 46% reported disruption of sleep for more than 4 hours. For non-confirmed coughing household contacts there were a similar number of coughing spells per day at the height, though the cough was reported to be less severe and to cause less sleep disruption. The main clinical symptoms on the worst day for both were shortness of breath, tiredness, sore ribs and vomiting. The duration of symptoms for both patient groups was around 160 days (162 and 168 days). Under base case assumptions the overall loss of quality of life was 0.097 QALY (0.089–0.106) for confirmed pertussis cases and 0.0365 QALY (0.023–0.054) for coughing household contacts.

**Conclusion:**

Pertussis is a serious disease in those aged 5 years and over, causing disruption of sleep and daily activities over long period of time. The burden of illness due to undiagnosed pertussis is also considerable.

## Background

Pertussis is a highly infectious bacterial disease that characteristically results in a prolonged spasmodic cough, often followed by vomiting or a high-pitched whooping sound as air is inhaled after the paroxysm. In young infants the illness is clinically severe, frequently resulting in hospital admission and sometimes death. However, in older children and adults the disease burden is more difficult to quantify as the symptoms are less clear and can be of a milder nature.

In the 1950s pertussis vaccines were introduced into the national childhood immunisation programme of many developed countries and, where coverage was high, were successful in controlling disease. However, in the last decade an increasing number of countries have observed pertussis outbreaks and report a resurgence in pertussis [1]. As the main aim of vaccination is to reduce morbidity and mortality in young infants, additional vaccination strategies are now being considered to protect this vulnerable age group. These strategies include vaccination of new-borns, vaccination of pregnant and lactating mothers, vaccination of adolescents and adults and “cocooning” which involves the vaccination of close family contacts of the neonate [1].

To enhance rational and transparent decision making, possible interventions are frequently compared in a cost-effectiveness analysis. This requires quantification of disease burden in terms of health related quality of life measures. To date only one published paper has attempted this for pertussis [2]. The authors used a method where putative pertussis-related disease states were evaluated by a panel of patients who had previously had pertussis. However, this approach has the major limitation that no actual disease states of patients were evaluated. Actual diseases seldom conform to standard clinical descriptions and therefore these estimates may not reflect reality.

In 2011–2012 a large pertussis outbreak occurred in England, with the highest incidence for over a decade and 16 related deaths [3]. This outbreak provided an opportunity to estimate the disease burden in those aged 5 years and older and obtain health related quality of life estimates for cost-effectiveness modelling. Pertussis is likely to be under reported [4], therefore we tried to capture both confirmed disease and non-confirmed disease. We performed a study in which patients with laboratory-confirmed pertussis, or household contacts of these cases who had a cough but no laboratory confirmation of pertussis, were asked to complete the 5 dimensional EuroQol questionnaire (EQ-5D) [5]. The EQ-5D, in combination with information on illness duration, can provide an estimate of the overall reduction in quality of life associated with a disease episode. We also measured utilisation of health care, drug use, time off work, and documented clinical symptoms in the study population.

## Methods

Cases of pertussis in patients aged 5 years and over in England and Wales who met the following criteria were included in the study: positive culture for *Bordetella pertussis*, or a positive serology test by the Bordetella Reference Laboratory at Public Health England (PHE); and where written permission from the treating General Practitioner (GP) to send the patient a questionnaire was obtained. Information on dates of pertussis vaccination were obtained from the GP. Confirmed cases were asked to report household contacts with symptoms of pertussis; these household contacts were also included as a proxy for non-confirmed pertussis in order to collect information on disease that is not reported.

All patients meeting the inclusion criteria between September 2011 and January 2013 were sent a questionnaire and a prepaid envelope to return completed forms. The questionnaire had three sections; the first collected background information on sex, age, vaccine history, date of onset, health care use, antibiotic use, hospitalisation, and time off work/school due to the pertussis infection. The second section focussed on disease severity on the day they filled in the questionnaire including information on the cough (number of cough spells, cough severity, and whether or not the cough kept them awake) and any other reported symptoms, as well a quality of life questionnaire. The quality of life was investigated using questionnaires developed by EuroQol, which is age dependent; the EQ-5D-Y by proxy for those aged 5–7, a EQ-5D-Y for those aged 7–11 and a EQ-5D-3L questionnaire for those aged 12 and above. Each version included a Visual Analog Score (VAS). The third section was similar to the second section, but focussed on the worst day of the disease. The questionnaires are available on request.

According to the guidelines of the National Research Ethics Service [6] collection of quality of life information from patients comes within the remit of the enhanced surveillance activities of Public Health England (PHE) and is thus deemed service evaluation rather than research for which ethics approval is not required. Participants were aware that obtained information would inform clinical practice and public health decisions. The data was anonymised before analysis.

Responders were followed-up every two to four weeks for as long as they reported pertussis- related symptoms to determine the duration of illness. To optimise the response rate the respondents were only required to answer “yes” or “no” to the question “Is your cough still affecting your usual health and/or sleep?” using their preferred method of communication for follow up; letter, text or phone. Therefore we only know between which dates the symptoms subsided (the last contact date and the date of the previous “yes” answer). The base case results are based on the midpoints between these two dates, but in the sensitivity analysis the outcomes using the minimum and the maximum duration of disease are shown.

Some of the laboratory confirmed cases were tested not because they visited the GP with symptoms, but as a part of an outbreak investigation, and could therefore be asymptomatic or have very mild symptoms. To measure the impact on the quality of life of symptomatic pertussis only we excluded laboratory confirmed cases who did not report pertussis-like symptoms on the worst day of their illness or who had not consulted a health professional. From the sample of non-confirmed pertussis we excluded those who were tested for pertussis (irrespective of test result) and those who did not cough on the worst day of disease.

The socio-economic status of responders and non-responders was based on the Index of Multiple Deprivation (2010) and assigned to residential postcode based on the Lower Super Output statistics, using the Lower Super Output Area boundaries of 2001.

### Calculating the loss in Quality Adjusted Life Years (QALY)

**Figure 1:**
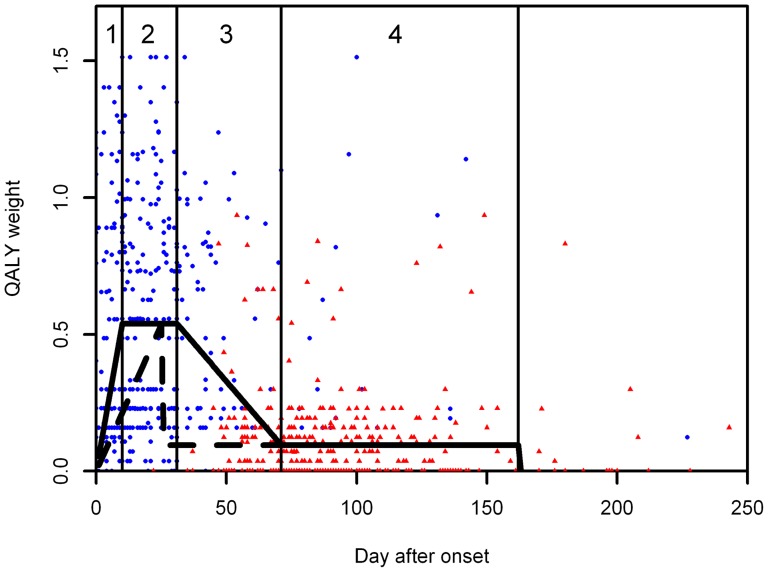
The data and the overall QALY loss as used in this analysis. The dots are the QALY weight by time after onset for the worst day (blue) and the day of the questionnaire (red). The black line represents the progression of disease severity over time, including the 4 stages (numbered 1,2,3 & 4), see text for an explanation. The dotted line shows the disease progression under the 2 stage approach.

To calculate the overall QALY loss the total area under the curve given the disease severity over time was used. Due to the relatively few data points we divided the disease course into four stages, described by six parameters (four different durations and two severity states). The first stage was the period between disease onset and the period of most severe symptoms, the second stage was the period during which the patient experienced the most severe symptoms followed by the third stage, a period where the symptoms improved but did not disappear. The fourth stage was a relatively long period where the patient remained symptomatic but the cough was relatively mild and slowly subsided. See (figure 1) for a graphical representation of the disease stages.

The level of the disease severity during the worst period (stage two) was informed by the mean quality of life on the worst day. The severity of the mild symptoms (stage four) was informed by the mean quality of life as measured on the day when the patient completed the questionnaire. Disease severity for stage one was assumed to increase linearly from onset to start of stage two, and for stage three, severity was assumed to decline linearly from the end of stage two to the start of stage four.

The duration of the stages was informed by the timing of the worst day, the timing after onset that the questionnaire was filled in and the duration of symptoms. The period of severe cough (stage two) was based on the inter quartile range of the timings of the worst day. The beginning of stage four was the lowest quartile of the day the questionnaire filled in. The end of stage four was the average total duration of having symptoms using a Kaplan-Meier survival curve to correct for censoring due to the patients lost to follow up. The method is fully described in supporting information S1.

The largest assumptions in the approach are the durations of the different disease states. Therefore the following sensitivity analysis was performed: we shortened the duration of the severe symptoms to one day (average day which was the most severe), and after the worst day there was no period of transitions to mild symptoms, but an immediate start of mild symptoms. In other words this eliminated stage two and three, and is therefore referred to as the 2-stage scenario. This approach should produce the lower boundary of the QALY loss estimate.

Data analysis was performed in R 3.02 using R studio. Confidence intervals were obtained by bootstrapping the original data (1000 samples).

## Results

### Response

The questionnaire was sent to 1262 laboratory confirmed pertussis patients aged between 6 and 90 years and 140 of their coughing household contacts aged between 7 and 74 years (Table 1). Of the laboratory confirmed cases 576 responded (48% response rate) of whom 41 met the exclusion criteria leaving 535 (42%) for the analysis. Of these 37% were reported by their GP to have received at least 3 doses of pertussis vaccine prior to onset. All but three were confirmed by serology (single high titre antibodies to pertussis toxin). For the household contacts there were 63 responders (45% response rate) of whom 19 were excluded, leaving 44. Based on the post code there were more patients from deprived areas compared to affluent areas. The response by age varied little, with the lowest response rate among adolescents and young adults: 44%, 33%, 46% and 47% for those aged 5–15,16–24,35–64 and 65+ respectively. There were relatively more children among the household contacts compared to the laboratory confirmed cases (Table 1). The questionnaire was filled in at a mean of 94 days after onset (range 22–243) for the laboratory confirmed cases and a mean of 105 days (range 42–189) for coughing household contacts. For 115 of the 535 (21%) confirmed cases and for 23 of the 44 (52%) coughing household contacts symptoms had stopped by the time of the questionnaire.

**Table 1:** Overview of the total approached and those who responded.

	Laboratory confirmed cases		Household contacts	
	Sent	Responded and included (% responded; % of the sample)	Sent	Responded and included (% responded; % of the sample)
**Total**	**1262**	**535 (42%∶100%)**	**140**	**44 (31%∶100%)**
5–15 yrs	197	87 (44%∶16%)	23	18 (78%∶41%)
16–24 yrs	146	48 (33%∶9%)	7	4 (57%∶9%)
25–64 yrs	808	373 (46%∶70%)	31	20 (65%∶45%)
65 yrs and over	55	26 (47%∶5%)	3	2 (67%∶5%)
Unknown	56	1 (2%∶0%)	76	0 (0%∶0%)
Sex (female)	NK	307 (57%)	NK	25 (57%)
Vaccination status confirmed by the GP		532	Not requested	
3 or more doses	Median age: 21	196 (37%)		
1 or 2 doses	Median age: 32	33 (6%)		
Not vaccinated	Median age: 44	68 (13%)		
Unknown vaccination status	Median age: 46	235 (44%)		
Socio-economic status				
20% most affluent	110 (9%)	40 (7%)		
20% less affluent	200 (17%)	78 (14%)		
20% middle	241 (20%)	113 (20%)		
20% more deprived	305 (25%)	146 (26%)		
20% most deprived	348 (29%)	175 (32%)		
	1204 (100%)	552 (100%)		

### Timing of worst day

Most patients timed the worst day as 2 to 4 weeks after onset (Table 2).

**Table 2:** Timing of the worst day.

Weeks after onset	Laboratory confirmed cases	Household contacts
Day of onset	7 (3%)	0 (0%)
1^st^ week	44 (18%)	3 (20%)
2^nd^ week	64 (26%)	4 (27%)
3^rd^ week	43 (17%)	2 (13%)
4^th^ week	30 (12%)	5 (33%)
5^th^ week	26 (11%)	0 (0%)
6^th^ week	13 (5%)	0 (0%)
7^th^ week	7 (3%)	1 (7%)
8^th^ week or over	13 (5%)	0 (0%)
	247	15

### Severity of cough on the worst day

For laboratory confirmed cases the cough on the worst day was characterised by many (20+ per day) coughing spells (56%), mainly very severe cough (58%), affecting the sleep for more than 4 hours (46%), see table 3. For household contacts there was an equal number of coughing spells, but the cough was reported to be less severe and affected sleep less (p<0.05 for the worst two categories, Chi-square test).

**Table 3:** Patient's assessment of the severity of cough on the worst day and the day of the questionnaire (later day) measured by the number of paroxysms, self assigned severity and duration of sleep disturbance.

	Laboratory confirmed cases						Household contacts					
	Coughing spells		Cough severity		Affects sleep		Coughing spells		Cough severity		Affects sleep	
Spells/severity/sleep	Worst day	Later day	Worst day	Later day	Worst day	Later day	Worst day	Later day	Worst day	Later day	Worst day	Later day
0–1/very mild/not at all	1 (0%)	65 (17%)	1 (0%)	73 (19%)	8 (2%)	252 (66%)	0 (0%)	12 (35%)	0 (0%)	12 (39%)	5 (11%)	23 (70%)
2–5/mild/20 min–1 hr	13 (2%)	174 (45%)	3 (1%)	119 (32%)	55 (10%)	96 (25%)	1 (2%)	14 (41%)	2 (5%)	7 (23%)	11 (25%)	9 (27%)
6–10/moderate/1–2 hrs	50 (9%)	85 (22%)	29 (5%)	150 (40%)	88 (17%)	20 (5%)	8 (18%)	5 (15%)	11 (25%)	11 (35%)	10 (23%)	1 (3%)
11–20/severe/2–4 hrs	172 (32%)	44 (11%)	193 (36%)	30 (8%)	136 (26%)	8 (2%)	13 (30%)	2 (6%)	16 (36%)	1 (3%)	7 (16%)	0 (0%)
20+/very severe/4+hrs	295 (56%)	17 (4%)	307 (58%)	3 (1%)	241 (46%)	6 (2%)	22 (50%)	1 (3%)	15 (34%)	0 (0%)	11 (25%)	0 (0%)
Responders who answered the question	530	385	533	375	528	382	44	34	44	31	44	33

### Clinical symptoms on the worst day

The main clinical symptoms on the worst day for both confirmed cases and household contacts were shortness of breath, tiredness, sore ribs and vomiting (table 4). The household contacts had fewer symptoms per patient.

**Table 4:** Clinical symptoms on the worst day and on the day the patients completed the questionnaire.

	Laboratory confirmed cases		Household contacts	
	Worst day	Day of the questionnaire	Worst day	Day of the questionnaire
No additional clinical symptoms	0	36 (8%)	1 (2%)	7 (19%)
Short of breath after cough	444 (84%)	239 (50%)	28 (64%)	7 (19%)
Tiredness	416 (79%)	202 (43%)	32 (73%)	10 (28%)
Cough with ‘whoop’	398 (75%)	120 (25%)	30 (68%)	5 (14%)
Sore ribs after cough	327 (62%)	104 (22%)	18 (41%)	2 (6%)
Vomiting after cough	321 (61%)	68 (14%)	22 (50%)	2 (6%)
Sore throat	310 (59%)	107 (23%)	22 (64%)	3 (8%)
Headache	297 (56%)	101 (21%)	20 (45%)	4 (11%)
Blood shot eyes	189 (36%)	63 (13%)	6 (14%)	3 (8%)
Weight loss	96 (18%)	28 (6%)	4 (9%)	1 (3%)
Pneumonia	49 (9%)	3 (1%)	1 (2%)	1 (3%)
Responders who answered the question	529 (5.5 symptoms per patient)	475 (2.4 symptoms per patient)	44 (4.2 symptoms per patient)	36 (1.3 symptoms per patient)

### Quality of life on the worst day

The dimensions most affected for laboratory confirmed contacts were ‘anxiety’ (17% severely affected) and ‘mobility’ (14% severely affected). In contrast, the most affected dimensions for the coughing household contacts were ‘usual activities’ and ‘pain/discomfort’ with 16% and 9% each severely affected respectively, see table 5.

**Table 5:** Scores for the individual 5 dimensions of the EQ-5D for the worst day and the day of the questionnaire.

	Worst day				Day of the questionnaire			
*Laboratory confirmed cases*	N	No problems	Some problems	Severe problems	N	No problems	Some problems	Severe problems
Self care	521	421 (81%)	80 (15%)	20 (4%)	418	409 (98%)	8 (2%)	1 (0%)
Mobility	523	245 (46%)	205 (39%)	73 (14%)	416	385 (93%)	30 (7%)	1 (0%)
Usual activities	526	269 (51%)	204 (39%)	53 (10%)	418	288 (69%)	120 (29%)	10 (2%)
Pain/discomfort	524	327 (62%)	149 (28%)	48 (9%)	415	280 (67%)	122 (29%)	13 (3%)
Anxiety depression	463	204 (44%)	182 (39%)	77 (17%)	414	317 (77%)	91 (22%)	6 (1%)
*Household contacts*								
Self care	44	43 (98%)	1 (2%)	0 (0%)	27	27 (100%)	0 (0%)	0 (0%)
Mobility	44	35 (80%)	9 (20%)	0 (0%)	27	25 (93%)	2 (7%)	0 (0%)
Usual activities	44	18 (41%)	19 (43%)	7 (16%)	27	20 (74%)	7 (26%)	0 (0%)
Pain/discomfort	43	16 (37%)	23 (53%)	4 (9%)	27	20 (74%)	7 (26%)	0 (0%)
Anxiety depression	41	30 (73%)	11 (27%)	0 (0%)	27	26 (96%)	1 (4%)	0 (0%)

For laboratory confirmed patients the average QALY weight based on the EQ-5D for the worst day of disease was 0.46 with a median of 0.54 and a minimum and maximum of −0.513 and 1. Unfortunately for 81 patients there was one or more missing answers in the EQ-5D. The average QALY weight based on the VAS was 35, with a median of 30 and minimum and maximum of 0 and 100; the VAS only had 19 missing values.

The coughing household contacts had an average QALY weight for the worst day of 0.80 with a median of 0.88 and maximum and minimum of 0.007 to 1. There were only 4 patients with missing information. Using the VAS, the median and the average were the same at 50 with a range of 15 to 100. There were no missing values for the VAS.

### Disease severity at a later stage

Although the timing after onset was not the same for each patient, by the day of the questionnaire cough severity had lessened (only 4% 20+ paroxysms a day), was milder in nature (only 1% very severe) and affected sleep less (2% affecting 4+hrs) in both laboratory confirmed cases and household contacts, see (table 3).

Overall patients had fewer clinical symptoms (see table 4); the biggest decline was in the most severe clinical symptoms such as pneumonia and bloodshot eyes; in 25% of confirmed cases, and 14% of household contacts a ‘whoop’ was still present.

### The quality of life on the day of the questionnaire

Of the five dimensions included in the EQ-5D, usual activities and pain and discomfort were the most affected on the day of the questionnaire, see table 5. In both confirmed cases and household contacts over 25% had one or more dimensions still affected. The QALY weight on the day of the questionnaire was 0.89 (0.065–1) for the laboratory confirmed cases and 0.9244 (0.70–1) for the household contacts. However the patient received the questionnaire at different times after disease onset. To test if there was a relation between time and QALY weight a linear regression model was fitted. For both the confirmed cases and the household contacts the factor for the slope was non-significant though the relation was positive (the later after onset the higher the QALY weight).

### Duration of symptoms

The duration of symptoms was estimated with a Kaplan-Meier survival curve. This was because despite a high response rate on our follow-up questionnaire in over 80% there was a significant drop out due to the long term follow-up, as patients answered up-to 16 times (maximum for any patient) that they still had symptoms.

There are three estimates of the duration; the mid and the minimum and maximum. For laboratory confirmed cases this was 162 days (minimum 152–maximum 174) for the household contacts 168 days (minimum 155–maximum 182).

### Health care seeking behaviour

Health care advice was sought by all confirmed cases and some had multiple forms of contact. Not all visited the GP, as some went to the A&E or other sources of care. Of the non-confirmed coughing household contacts 30% did not seek any health care advice. The average cost of care based on the survey was estimated to be £55.55 for confirmed cases and £25.63 for the household contacts (including those who did not seek care). Of the laboratory confirmed cases 60% took time off school or work, with a median duration of 7 days, for coughing household members only 41% took time off school or work with a median of 4.5 days (Table 6).

**Table 6:** Health care use* and time off work.

	Laboratory confirmed cases	Household contacts
None	0 (0%)	13 (30%)
Visits GP (£36 [11])	499 (93%)	25 (57%)
Call receptionist (£22 [11])	68 (13%)	3 (7%)
Call GP (£22 [11])	116 (22%)	9 (20%)
Call NHS direct (£27.42 [12])	62 (12%)	3 (7%)
Visits Hospital/A&E (£32 [11])	84 (16%)	3 (7%)
Other (£36 same as GP visit)	44 (8%)	1 (2%)
Average overall costs	£55.55 (SD 1.594)	£25.63 (SD 4.81)
Time off work/school	507 filled in the question	39 filled in the question
No time off work/school	204 (40%; 42 indicated a reason e.g. being retired or it was holiday)	23 (59%; 2 indicated an excuse)
Yes	303 (60%; median:7 days, mean:13 days, min-max: 1–80 days)	16 (41%; median:4.5 days, mean:4.3 days, min-max:0.5–10)

*Each patient can have multiple health care visits.

### Quality of life and cough severity

**Figure 2:**
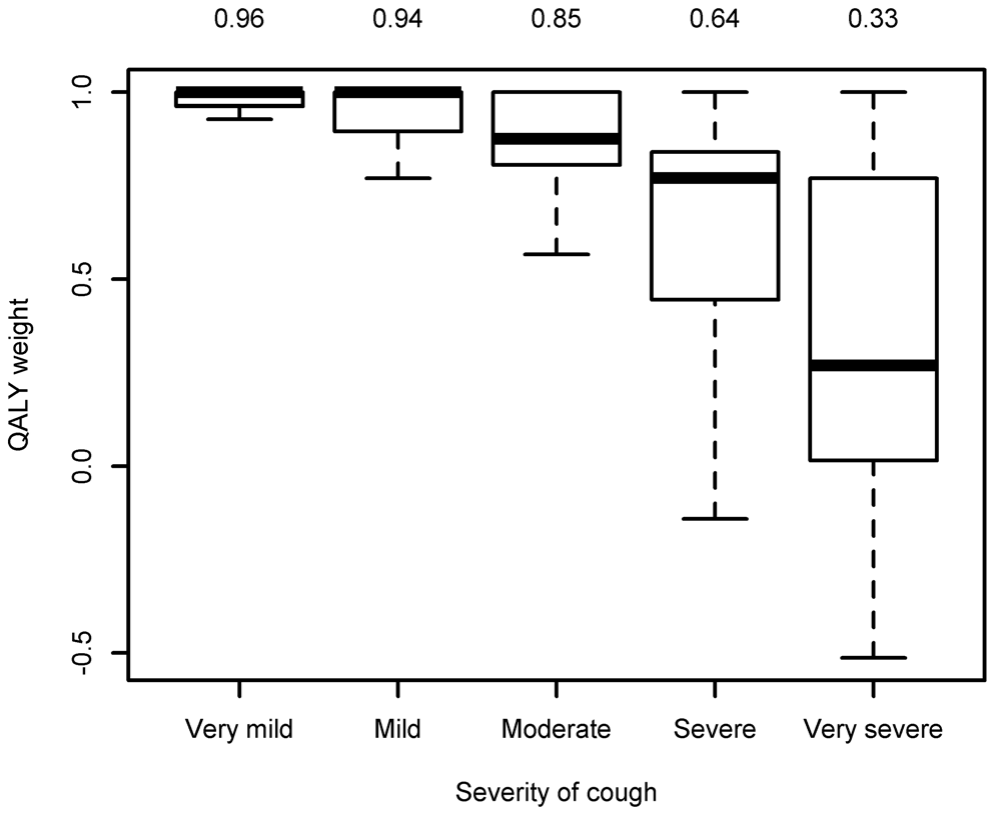
The boxplot of the QALY weight split by the severity of cough for laboratory confirmed cases with pooled data from both the worst day and the day of the questionnaire. Above the plot the mean values are presented.

Pooling the data of the laboratory cases for both the worst day and the day of the questionnaire shows that there was a relation between the measured quality of life detriment and the severity of the cough (Figure 2). The QALY weight decreased from an average of 0.96 for a very mild cough to 0.33 for a very severe cough. There was however a great variety in QALY weight scores, spanning 1 to −0.5 for a severe cough.

### Loss of quality of life

The overall loss of quality of life is presented in table 7 and was 0.097 QALY (0.089–0.106) for confirmed pertussis cases using the four stage approach, with the two stage approach it was 0.055 QALY (0.048–0.062). Coughing household contacts had a lower loss of quality of life with 0.0365 QALY (0.023–0.054) for the four stage approach and 0.020 QALY (0.012–0.032) for the two stage approach. Expressed in quality adjusted life days (QALY*365 days) this is 35.5 QALD and 19.9 QALD for the two measurements for confirmed cases and 13.3 QALD and 7.5 QALD for household contacts.

**Table 7:** The overall QALY loss using the four stage approach or the two stage approach, the standard deviation of the mean overall QALY loss is shown in brackets.

	Laboratory confirmed cases		Household contacts	
	4 stages	2 stages	4 stages	2 stages
QALY loss base	0.09724 (0.0044)	0.05450 (0.0037)	0.03645 (0.00772)	0.02042 (0.00507)
QALY loss min	0.09453 (0.0043)	0.05179 (0.0035)	0.03500 (0.00747)	0.01870 (0.00478)
QALY loss max	0.1002 (0.0055)	0.05758 (0.0038)	0.0380 (0.00797)	0.02196 (0.00533)

### Vaccination status

There were no differences in QALY loss, duration of cough, presence of vomiting or whooping in patients recorded as having received at least three doses of pertussis vaccine prior to onset compared with those who had not; even though those fully vaccinated were younger than those without.

## Discussion

Pertussis in young infants is recognised to be a severe illness, resulting in hospital admission and sometimes death. Our patient survey among those aged 5 and over reveals that pertussis causes a severe and prolonged cough, even in those who have been vaccinated in the past. In laboratory confirmed cases, a pertussis episode is associated with a QALY loss of around 0.055–0.097 or between 20 and 36 of quality adjusted life days. Pertussis in older age groups is therefore a serious disease. For example compared with influenza these scores are very high, as in confirmed influenza patients the overall loss in quality of life was estimated to be just under 3 days, almost 10 times less [7]. The main cause of the high QALY loss in pertussis is the long duration and severity of the cough, which affects sleep, induces vomiting and tiredness, and impacts the quality of life in dimensions such as mobility, daily activities and inducing anxiety/depression. For coughing household contacts the loss in quality of life, though lower, was still greater than that of an influenza episode. Interestingly the data suggests that the number of coughing spells and the duration of symptoms is not less in non-confirmed cases compared to confirmed cases, but that cough severity is less.

In all but three of the confirmed cases in our study laboratory diagnosis of pertussis was based on a single high antibody titre to pertussis toxin in a blood sample taken some weeks after onset, a method that has high specificity in patients with symptoms of pertussis infection [8]. Although many of the patients in our study had classic pertussis symptoms, as evidenced by a prolonged paroxysmal cough with vomiting and whooping, very few would have been confirmed as pertussis without the availability of serology as the diagnosis is rarely considered in adults until late in the course of the illness when culture and PCR methods are unlikely to yield positive results. While some of the increases in the serologically confirmed cases recently reported in the UK [9] may be due to increased awareness, it is clear that the endemic incidence of pertussis in adolescents and adults, even prior to the recent resurgence, is considerably higher than estimated from confirmed cases. One study of patients presenting in general practice in England with acute spasmodic cough of at least 3 weeks duration in 1996/7 estimated an incidence of serologically confirmed pertussis of 330 per 100,000, whereas statutory notifications of pertussis in England and Wales suggested an incidence of less than 4 per 100,000 in the same period [10]. Even patients with a prolonged cough accompanied by whooping may not seek health care or if they do, they may not be investigated for pertussis, as shown by our study in coughing household contacts of confirmed cases.

In the previous quality of life study on pertussis in the US [2] QALY weights were obtained for different pertussis-related health states as described to adults who had recovered from pertussis, or the parents of adolescent pertussis cases. The QALY weight obtained for mild cough was 0.87 for adolescents and 0.85 for adults and 0.67 and 0.81 respectively for severe cough. In comparison, the QALY weight for mild and severe cough was 0.94 and 0.64 respectively in our study. Although the studies used very different methodological approaches, it suggests that the mild cough in our study was in particular linked to less loss in quality of life, perhaps highlighting the drawbacks of measuring QALY weights for very mild disease states by time-trade-off methods as used in the US study.

Our study has a number of methodological limitations. First, there may be selection bias, as the patients with the most severe disease may be the most likely to respond, and keep responding during the follow-up. Another limitation is the lack of information on the exact duration of the various stages of the disease which necessitated assumptions about the time course, albeit based on the data provided by respondents. Another potential problem is recall bias given the long interval between onset and completion of the questionnaire. Also, the sample size for estimating QALY loss in non-confirmed cases was relatively small and consisted mainly of children. Due consideration needs to be given to these limitations when conducting analyses of the cost effectiveness of the different vaccination strategies that could reduce the current burden of morbidity and mortality from pertussis in the UK.

In conclusion, our study shows that the impact on the quality of life of confirmed pertussis disease in those aged 5 years and above is considerable when compared with other acute respiratory infections such as influenza. The overall burden of pertussis illness at a population level is likely to be considerably underestimated due to failure of patients with prolonged cough to seek medical care and under investigation of pertussis in those that do.

## Supporting Information

Supporting Information S1 (DOCX)
