# Supplementary material for: The Burden of Disease and Health Care Use among Pertussis Cases in School Aged Children and Adults in England and Wales; A Patient Survey
Source: PLoS One. 2014 Nov 25;9(11):e111807. doi: 10.1371/journal.pone.0111807 (PMC4244040; doi:10.1371/journal.pone.0111807)
Supplement: Supporting Information S1 — (DOCX) [file pone.0111807.s001.docx]

**S1 Supporting information: The calculation of the overall loss in quality of life.**

The overall loss in quality of life is the total area under the curve as shown in figure 1 of the main text. This curve was obtained via the following steps:

1. Set the five points in time

- Onset symptoms – day 1 by definition
- Start worst symptoms (α): 1^st^ quartile of the timing of the worst day
- End worst symptoms (β): 3^rd^ quartile of the timing of the worst day
- Start mild symptoms (µ): 1^st^ quartile of the timing of the day the participant filled in the questionnaire
- End symptoms (δ): the average total duration of disease as observed, controlling for the loss in follow-up. This was done by fitting a Kaplan-Meier curve to the overall duration and whether the person was lost to follow-up or had stopped being symptomatic.

2. The curve describing the worst symptoms

The curve is a straight line based on the mean quality of life detriment on the worst day.

$${QALY}_{i}^{worst}={mean QALY loss}^{worst}$$

3. The curve describing the milder symptoms

The curve describing the milder symptoms is a straight line based on the mean quality of life detriment on the day of the questionnaire.

$${QALY}_{i}^{mild}={mean QALY loss}^{mild}$$

The overall curve is divided into four parts, and was obtained as follows;

First part:

$${QALY}_{1\leq i\leq\alpha}^{final curve}=i* \left( \frac{{QALY}_{\alpha}^{worst}}{\alpha} \right)$$

Second part:

$${QALY}_{\alpha\leq i\leq\beta}^{final curve}={mean QALY loss}^{worst}$$

Third part:

$${QALY}_{\beta\leq i\leq\mu}^{final curve}= \left( i*\frac{{QALY}_{\beta}^{worst}-{QALY}_{\mu}^{mild}}{(\mu-\beta)} \right)+{QALY}_{\mu}^{mild}$$

Fourth part:

$${QALY}_{\mu\leq i\leq\delta}^{final curve}={mean QALY loss}^{mild}$$

The four parts together describe the curve from onset to the symptoms end, or day 1 to day δ. The overall loss is the total area under the curve of 1-QALY loss, which is the sum of the individual days. To obtain the quality adjusted life year the total needs to be divided by 365.

Table A1 Mean values and confidence intervals as obtained for each parameter in the bootstrap (1000 samples)

| Parameter | Mean (95% CI) |
| --- | --- |
| α | 9.79 (9-11) |
| β | 30.9 (30-32) |
| µ | 71.2 (68-75) |
| δ | 161.9 (153-172) |

R-code used to perform the bootstrap

#################################################################

## Code to obtain a bootstrap sample for the quality of life #

#################################################################

survival_function <- function(Days,Stopt_ongoing){

prepare_data <- cbind(Days,Stopt_ongoing)

prepare_data <- data.frame(prepare_data)

names(prepare_data) <- c("Days","stop_ongoing")

prepare_data <- prepare_data[!is.na(prepare_data$Days),]

prepare_data <- prepare_data[!prepare_data$Days<0,]

test <- list(duration=prepare_data$Days,stopped=prepare_data$stop_ongoing)

testesr <- survfit(Surv(duration,stopped)~1,data=test)

outcome_survival <- capture.output(print(testesr,print.rmean=TRUE), file = "test2.txt")

mean_today_to_end <- scan("test2.txt",nmax=5,skip=3,quiet=T)[5]

list(fit=testesr,result=outcome_survival,lol=mean_today_to_end)

}

numb_runs <- 1000

bootstrap_results <- c(rep(NA,numb_runs))

bootstrap_results_min <- bootstrap_results

bootstrap_results_max <- bootstrap_results

alfa <- c(rep(NA,numb_runs))

beta <- c(rep(NA,numb_runs))

zeta <- c(rep(NA,numb_runs))

delta <- c(rep(NA,numb_runs))

intercept_worst <- c(rep(NA,numb_runs))

coefficient_worst <- c(rep(NA,numb_runs))

intercept_mild <- c(rep(NA,numb_runs))

coefficient_mild <- c(rep(NA,numb_runs))

bootstrap_results_alt <- c(rep(NA,numb_runs))

bootstrap_results_min_alt <- bootstrap_results

bootstrap_results_max_alt <- bootstrap_results

for(bootstrap in 1:numb_runs){

selector <- sample(step3$Patient_Number,length(step3$Patient_Number),replace=TRUE)

selection <- unlist(sapply(selector, function(x) which(x==step3$Patient_Number)))

step3_bootstrap <- step3[selection,]

duration_middle <- survival_function(step3_bootstrap$onset_endmiddle,step3_bootstrap$stopt_lost_ongoing)

duration_min <- survival_function(step3_bootstrap$onset_endminimum ,step3_bootstrap$stopt_lost_ongoing)

duration_max <- survival_function(step3_bootstrap$onset_endmaximum ,step3_bootstrap$stopt_lost_ongoing)

QALYloss_worst_day_funtion <- rep(mean(step3_bootstrap$QALYloss_worst_day,na.rm=T),duration_middle$lol)

QALYloss_today_function <- rep(mean(step3_bootstrap$QALYloss_today,na.rm=T),duration_max$lol)

Timing_worst_day <- summary(step3_bootstrap$onset_worst,na.rm=T)

Timing_today <- summary(step3_bootstrap$onset_today[is.na(step3_bootstrap$Date.Symptoms.end)],na.rm=T)

Start_QALY_loss_worst <- QALYloss_worst_day_funtion[Timing_worst_day[2]]

End_QALY_loss_worst <- QALYloss_worst_day_funtion[Timing_worst_day[5]]

stage1 <- c(1:Timing_worst_day[2])*(Start_QALY_loss_worst/Timing_worst_day[2])

stage2 <- QALYloss_worst_day_funtion[(Timing_worst_day[2]+1):(Timing_worst_day[5]-1)]

Start_QALY_loss_mild <- QALYloss_today_function[Timing_today[2]]

stage3 <- c((Timing_today[2]-Timing_worst_day[5]):1)*(End_QALY_loss_worst-Start_QALY_loss_mild)/(Timing_today[2]-Timing_worst_day[5])

stage3 <- stage3+Start_QALY_loss_mild

stage4 <- QALYloss_today_function[Timing_today[2]:round(duration_middle$lol)]

stage4_min <- QALYloss_today_function[Timing_today[2]:round(duration_min$lol)]

stage4_max <- QALYloss_today_function[Timing_today[2]:round(duration_max$lol)]

overall <- c(stage1,stage2,stage3,stage4)

overall_min <- c(stage1,stage2,stage3,stage4_min)

overall_max <- c(stage1,stage2,stage3,stage4_max)

bootstrap_results[bootstrap] <- sum(overall)/365

bootstrap_results_min[bootstrap] <- sum(overall_min)/365

bootstrap_results_max[bootstrap] <- sum(overall_max)/365

Start_QALY_loss_worst_alt <- QALYloss_worst_day_funtion[Timing_worst_day[4]]

stage1_alt <- c(1:Timing_worst_day[4])*(Start_QALY_loss_worst_alt/Timing_worst_day[4])

stage4_alt <- QALYloss_today_function[Timing_worst_day[4]:round(duration_middle$lol)]

stage4_min_alt <- QALYloss_today_function[Timing_worst_day[4]:round(duration_min$lol)]

stage4_max_alt <- QALYloss_today_function[Timing_worst_day[4]:round(duration_max$lol)]

overall_alt <- c(stage1_alt,stage4_alt)

overall_min_alt <- c(stage1_alt,stage4_min_alt)

overall_max_alt <- c(stage1_alt,stage4_max_alt)

bootstrap_results_alt[bootstrap] <- sum(overall_alt)/365

bootstrap_results_min_alt[bootstrap] <- sum(overall_min_alt)/365

bootstrap_results_max_alt[bootstrap] <- sum(overall_max_alt)/365

alfa[bootstrap] <- Timing_worst_day[2]

beta[bootstrap] <- Timing_worst_day[5]

zeta[bootstrap] <- Timing_today[2]

delta[bootstrap] <- round(duration_middle$lol)

aver_worst[bootstrap] <- mean(step3_bootstrap$QALYloss_worst_day,na.rm=T)

aver_mild[bootstrap] <- mean(step3_bootstrap$QALYloss_today,na.rm=T)

print(bootstrap)

}
